# Ameliorative Effects of *Lactobacillus plantarum* HAC01 Lysate on 3T3-L1 Adipocyte Differentiation via AMPK Activation and MAPK Inhibition

**DOI:** 10.3390/ijms23115901

**Published:** 2022-05-24

**Authors:** Jong-Yeon Kim, Eun-Jung Park, Hae-Jeung Lee

**Affiliations:** 1 Department of Food Science and Biotechnology, Gachon University, Seongnam 13120, Gyeonggi-do, Korea; whddus95@gachon.ac.kr; 2 Department of Food and Nutrition, Gachon University, Seongnam 13120, Gyeonggi-do, Korea; 3 Institute for Aging and Clinical Nutrition Research, Gachon University, Seongnam 13120, Gyeonggi-do, Korea

**Keywords:** *Lactobacillus plantarum* HAC01, anti-obesity, lipid metabolism, AMPK, MAPK

## Abstract

*Lactobacillus plantarum* HAC01 has been shown to effectively treat metabolic diseases. However, the precise pharmacological effects and molecular mechanisms of *L. plantarum* HAC01 remain unclear. In this study, we investigate the anti-adipogenic effects of *L. plantarum* HAC01 lysate and its associated mechanism of action. To induce lipid accumulation, 3T3-L1 cells were incubated in differentiation media with or without *L. plantarum* HAC01 lysate. Our results show that *L. plantarum* HAC01 lysate treatment not only reduced lipid accumulation during the differentiation of 3T3-L1 cells, but also decreased the expression of adipogenic and lipogenic genes involved in lipid metabolism in a dose-dependent manner. Additionally, *L. plantarum* HAC01 lysate inhibited CCAAT/enhancer-binding protein (C/EBP) beta within 4 h of differentiation induction and inhibited peroxisome proliferator-activated receptor gamma, C/EBP alpha, and sterol regulatory element-binding proteins within 2 d. Moreover, treatment with *L. plantarum* HAC01 lysate increased the phosphorylation of adenosine monophosphate-activated protein kinase, an important regulator of energy metabolism, and decreased the phosphorylation of mitogen-activated protein kinase. These results indicate that *L. plantarum* HAC01 lysate may have anti-adipogenic effects and support its potential as a useful agent for the treatment of obesity.

## 1. Introduction

The excessive accumulation of body fat leads to obesity, which increases the risk of non-communicable diseases, such as type-2 diabetes and cardiovascular diseases [1,2]. Therefore, many methods have been proposed to reduce body fat accumulation. The most common solution is exercise; however, the intake of natural products or chemicals with anti-obesity effects has also become popular [3]. Various natural products that can reduce body fat accumulation have been studied, among which probiotics are known to exert anti-obesity effects [3,4].

Probiotics are a type of viable microorganism that primarily maintain beneficial bacteria in the gut and suppress the growth of harmful bacteria. *Lactobacillus* and *Bifidobacterium* are widely regarded as safe probiotics with many potential health benefits, including the amelioration of obesity, metabolic syndromes, immune disorders, and irritable bowel syndrome [5]. Owing to concerns about the safety and usefulness of viable probiotics, probiotic lysates have recently gained attention as an alternative [6]. Both probiotics and probiotic lysates have demonstrated beneficial effects on various diseases and conditions, such as colitis, inflammation, and tumors [7,8]. Interestingly, several studies have shown that probiotic lysates ameliorate obesity by decreasing body weight, especially adipose tissue weight, lowering total cholesterol, and reducing the expression of adipogenic factors in vitro and in vivo [9,10,11].

Adipogenesis is the process of differentiation from pre-adipocytes to mature adipocytes, accompanied by changes in gene expression and cell morphology [12]. Various transcription factors, including CCAAT/enhancer-binding proteins beta and alpha (C/EBPβ and α), peroxisome proliferator-activated receptor-gamma (PPARγ), and sterol regulatory element-binding protein-1c (SREBP-1c), regulate the early stage of adipocyte differentiation [13]. These transcription factors are suppressed by activated adenosine monophosphate-activated protein kinase (AMPK), a regulatory sensor of cellular energy metabolism [14]. Furthermore, mitogen-activated protein kinase (MAPK) signaling, which involves extracellular regulated kinase (ERK), p38, and c-Jun N-terminal kinase (JNK), plays a critical role in many cellular processes, including adipocyte differentiation [15].

Studies have shown that *Lactobacillus plantarum* HAC01 isolated from Korean white kimchi (baek kimchi) positively affects weight loss, hyperlipidemia, and type-2 diabetes in high-fat diet mice [16,17,18]. In addition, it has been demonstrated that *L. plantarum* HAC01 can be used as a beneficial modulator of gut microbiota [16]. However, the molecular mechanism underlying the effects of *L. plantarum* HAC01 lysate on obesity remains largely unknown. In this study, we aim to evaluate the anti-obesity effects of *L. plantarum* HAC01 lysate on 3T3-L1 pre-adipocytes and investigated its effects on the AMPK and MAPK signaling pathways to gain insight into the underlying mechanisms.

## 2. Results

### 2.1. L. plantarum HAC01 Lysate Inhibits the Differentiation of 3T3-L1 Cells

**Figure 1:**
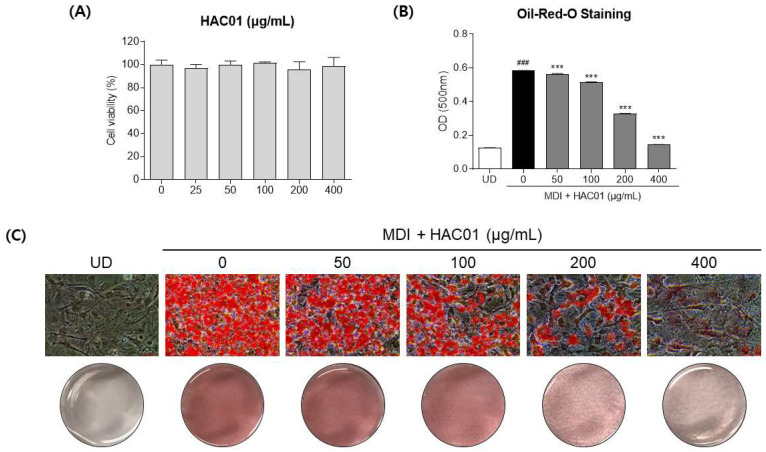
Effects of *Lactobacillus plantarum* HAC01 lysate on lipid accumulation in 3T3-L1 cells. (**A**). Cell viability. (**B**) Quantification of Oil Red O-stained lipid droplets. (**C**) Representative Oil Red O staining images. Images were captured at 200× magnification. The scale bar indicates 50 μm. OD, optical density; UD, undifferentiated; MDI, 500 μM 3-isobutyl-1-methylxanthine, 1 μM dexamethasone, and 10 μg/mL insulin. All results are presented as mean ± standard deviation. ^###^ *p* < 0.001 vs. UD; *** *p* < 0.001 vs. 0 μg/mL.

We first conducted a CCK-8 assay to determine the non-cytotoxic concentration of *L. plantarum* HAC01 lysate. The results demonstrate that *L. plantarum* HAC01 lysate was not cytotoxic to 3T3-L1 pre-adipocyte cells at concentrations ranging from 25 to 400 μg/mL (Figure 1A); thus, these concentrations were used in our experiments.

To assess the effects of *L. plantarum* HAC01 lysate on lipid accumulation, adipocyte differentiation was induced in 3T3-L1 cells for 7 d, followed by staining with Oil Red O solution (Figure 1B,C). Differentiated 3T3-L1 cells displayed a significant increase in lipid accumulation compared to undifferentiated cells, whereas *L. plantarum* HAC01 lysate-treated cells exhibited a significant dose-dependent decrease in lipid accumulation. In particular, lipid droplets were visibly diminished at a lysate concentration of 400 μg/mL. These results indicate that *L. plantarum* HAC01 lysate can effectively reduce adipocyte differentiation and lipid accumulation.

### 2.2. L. plantarum HAC01 Lysate Inhibits the Expression of Adipogenic and Lipogenic Factors in 3T3-L1 Pre-Adipocytes

**Figure 2:**
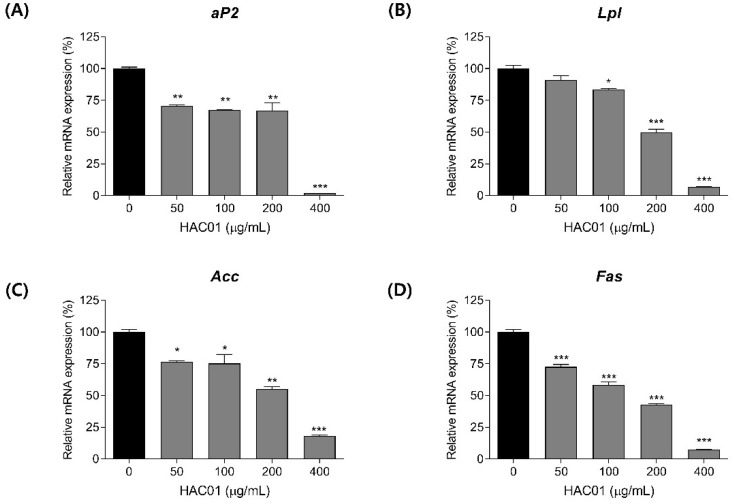
Effects of *Lactobacillus plantarum* HAC01 lysate on the mRNA expression levels of adipogenic and lipogenic factors in 3T3-L1 cells. Adipocyte differentiation was induced in the presence of *L. plantarum* HAC01 lysate for 7 d. mRNA expression levels of (**A**) *aP2*, (**B**) *Lpl*, (**C**) *Acc*, and (**D**) *Fas* were quantified using qRT-PCR. *aP2*, adipocyte protein 2; *Lpl*, lipoprotein lipase; *Acc*, acetyl-CoA carboxylase; *Fas*, fatty acid synthase. All results are presented as mean ± SD. * *p* < 0.01, ** *p* < 0.005, and *** *p* < 0.001 vs. 0 μg/mL.

**Figure 3:**
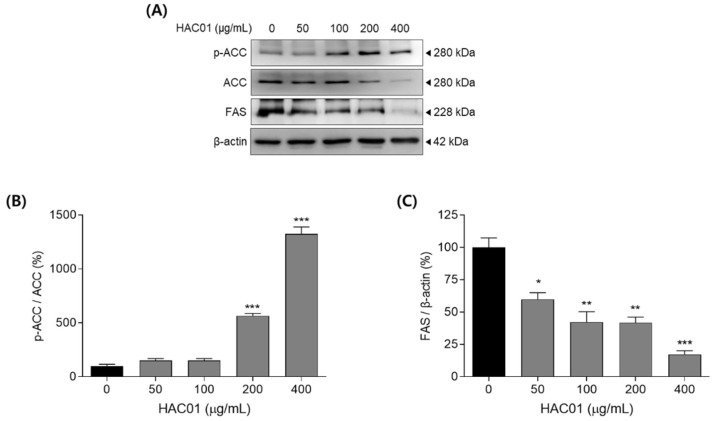
Effects of *Lactobacillus plantarum* HAC01 lysate on the protein expression levels of lipogenic factors in 3T3-L1 cells. Adipocyte differentiation was induced in the presence of *L. plantarum* HAC01 lysate for 7 d. (**A**) Western blot band images of lipogenic factors. Representative bands from triplicate experiments are shown. (**B**,**C**) Relative protein expression levels of (**B**) p-ACC/ACC and (**C**) FAS/β-actin. ACC, acetyl-CoA carboxylase; FAS, fatty acid synthase. All results are presented as mean ± SD. * *p* < 0.01, ** *p* < 0.005, and *** *p* < 0.001 vs. 0 μg/mL.

After differentiation, pre-adipocytes become mature adipocytes and turn on the expression of adipogenic and lipogenic factors. We examined whether *L. plantarum* HAC01 lysate inhibited the expression of adipogenic and lipid synthesis markers on day 7 after differentiation. Cells treated with *L. plantarum* HAC01 lysate exhibited significantly reduced mRNA expression levels of adipocyte protein 2 (aP2), lipoprotein lipase (Lpl), acetyl-CoA carboxylase (Acc), and fatty acid synthase (Fas) in a dose-dependent manner (Figure 2). Moreover, *L. plantarum* HAC01 lysate treatment activated ACC phosphorylation and reduced FAS protein levels (Figure 3).

### 2.3. L. plantarum HAC01 Lysate Suppresses the Early Stage of Adipocyte Differentiation

**Figure 4:**
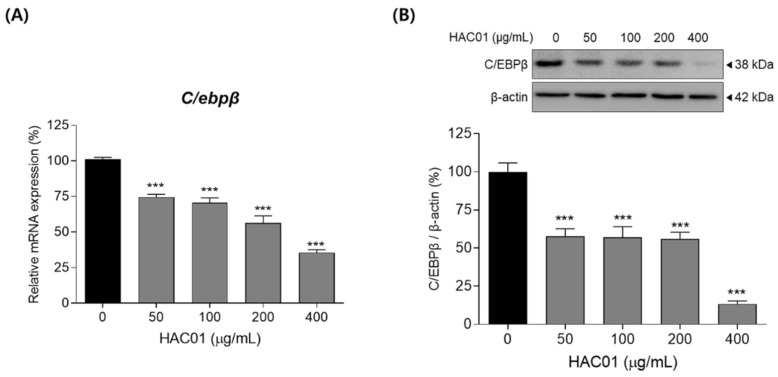
Effects of *Lactobacillus plantarum* HAC01 lysate on the expression of C/EBPβ in 3T3-L1 cells. Adipocyte differentiation was induced in the presence of *L. plantarum* HAC01 lysate for 4 h. (**A**) mRNA expression of *C/ebpβ* quantified using qRT-PCR. (**B**) Protein expression levels of C/EBPβ compared to β-actin quantified using Western blotting. Representative bands from triplicate experiments are shown. C/EBPβ, CCAAT/enhancer-binding protein beta. All results are presented as mean ± SD. *** *p* < 0.001 vs. 0 μg/mL.

To identify whether *L. plantarum* HAC01 lysate suppresses the early stage of adipocyte differentiation, we investigated the expression of C/EBPβ in 3T3-L1 cells after 4 h of differentiation. Treatment with *L. plantarum* HAC01 lysate significantly suppressed the mRNA and protein levels of C/EBPβ (Figure 4).

**Figure 5:**
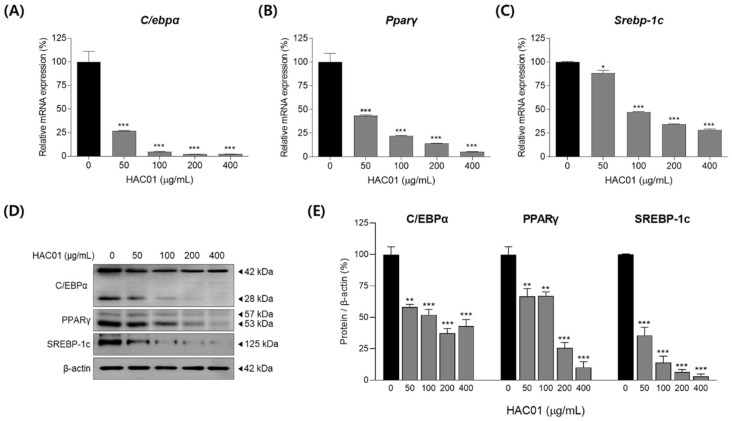
Effects of *Lactobacillus plantarum* HAC01 lysate on the expression of transcription factors during the early stage (2 d) of adipocyte differentiation in 3T3-L1 cells. Adipocyte differentiation was induced in the presence of *L. plantarum* HAC01 lysate for 2 d. mRNA expression levels of (**A**) *C/ebpα*, (**B**) *Pparγ*, and (**C**) *Srebp-1c* were quantified using qRT-PCR. (**D**) Western blot band images of transcription factors. Representative bands from triplicate experiments are shown. (**E**) Protein expression levels of C/EBPα (42 and 28 kDa), PPARγ (53 and 57 kDa), and SREBP-1c quantified using Western blotting. C/EBPα, CCAAT/enhancer-binding protein alpha; PPARγ, peroxisome proliferator-activated receptor-gamma; SREBP-1c, sterol regulatory element-binding protein 1c. All results are presented as mean ± SD. * *p* < 0.01, ** *p* < 0.005, and *** *p* < 0.001 vs. 0 μg/mL.

**Figure 6:**
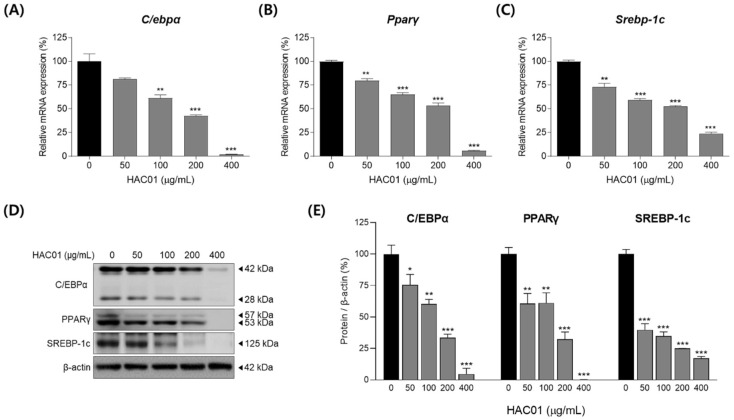
Prolonged effects of *Lactobacillus plantarum* HAC01 lysate on the expression of transcription factors in 3T3-L1 cells after 7 d of differentiation. Adipocyte differentiation was induced in the presence of *L. plantarum* HAC01 lysate for 7 d. (**A**–**C**) mRNA expression levels of (**A**) *C/ebpα*, (**B**) *Pparγ*, and (**C**) *Srebp-1c* were quantified using qRT-PCR. (**D**) Western blot band images of transcription factors. Representative bands from triplicate experiments are shown. (**E**) Protein expression levels of C/EBPα (42 and 28 kDa), PPARγ (53 and 57 kDa), and SREBP-1c quantified using Western blotting. C/EBPα, CCAAT/enhancer-binding protein alpha; PPARγ, peroxisome proliferator-activated receptor-gamma; SREBP-1c, sterol regulatory element-binding protein 1c. All results are presented as mean ± SD. * *p* < 0.01, ** *p* < 0.005, and *** *p* < 0.001 vs. 0 μg/mL.

We also examined the expression of transcription factors on day 2 of differentiation. The mRNA expression levels of *C/ebpα*, *Pparγ*, and *Srebp-1c* were significantly decreased following *L. plantarum* HAC01 lysate treatment (Figure 5A–C). Similarly, Western blotting demonstrated that the protein levels of C/EBPα, PPARγ, and SREBP-1c were also significantly reduced following treatment (Figure 5D,E). These effects of *L. plantarum* HAC01 lysate treatment were sustained for up to 7 d (Figure 6). Together, these results indicate that HAC01 lysate suppresses the early stage of adipocyte differentiation.

### 2.4. L. plantarum HAC01 Lysate Inhibits Adipocyte Differentiation via the Activation of AMPK

**Figure 7:**
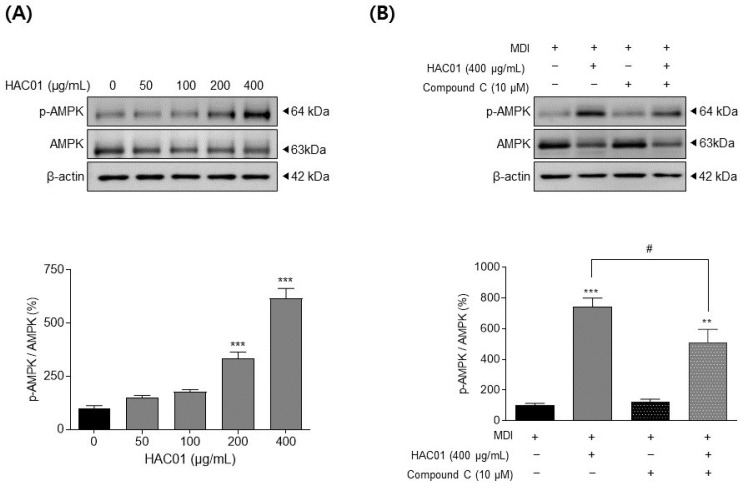
Effects of *Lactobacillus plantarum* HAC01 lysate on AMPK activation in 3T3-L1 cells. Adipocyte differentiation was induced in the presence of *L. plantarum* HAC01 lysate for 2 d. (**A**) Protein expression levels of *p*-AMPK/AMPK quantified using Western blotting. (**B**) Protein expression levels of *p*-AMPK/AMPK, with or without Compound C, quantified using Western blotting. Representative bands from triplicate experiments are shown. AMPK, adenosine monophosphate-activated protein kinase. All results are presented as mean ± SD. ** *p* < 0.005 and *** *p* < 0.001 vs. 0 μg/mL; # *p* < 0.005 vs. 400 μg/mL.

To evaluate the molecular mechanism underlying the anti-adipogenic properties of *L. plantarum* HAC01 lysate, we investigated the activation of AMPK, which suppressed adipocyte differentiation, during the early differentiation period (day 2). As shown in Figure 7A, the phospho-AMPK/AMPK ratio was elevated following treatment with *L. plantarum* HAC01 lysate, indicating increased pathway activity. This led us to hypothesize that *L. plantarum* HAC01 lysate inhibits adipocyte differentiation via the AMPK signaling pathway, which we tested by treating 3T3-L1 cells with *L. plantarum* HAC01 lysate and an AMPK inhibitor, Compound C. The observed increase in AMPK phosphorylation following *L. plantarum* HAC01 lysate treatment alone was significantly attenuated by co-treatment with Compound C (Figure 7B). These results suggest that *L. plantarum* HAC01 lysate exerts its inhibitory effect on early adipocyte differentiation via AMPK phosphorylation.

### 2.5. L. plantarum HAC01 Lysate Regulates the Phosphorylation of MAPK

**Figure 8:**
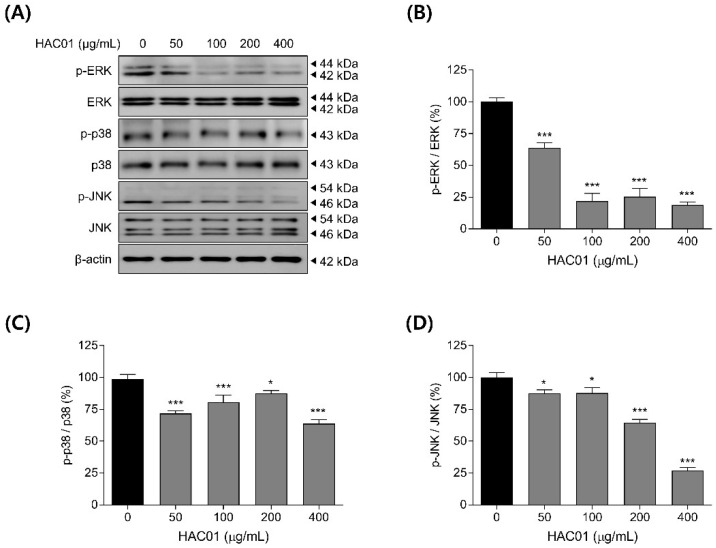
Effects of *Lactobacillus plantarum* HAC01 lysate on MAPK inhibition in 3T3-L1 cells. Adipocyte differentiation was induced in the presence of *L. plantarum* HAC01 lysate for 2 d. (**A**) Western blotting band images of MAPK signaling components. Representative bands from triplicate experiments are shown. (**B**–**D**) Relative protein expression levels of (**B**) *p*-ERK/ERK, (**C**) *p*-p38/p38, and (**D**) *p*-JNK/JNK quantified using Western blotting. ERK, extracellular regulated kinase; JNK, c-Jun N-terminal kinase. All results are presented as mean ± SD. * *p* < 0.01 and *** *p* < 0.001 vs. 0 μg/mL.

The inhibition of MAPK pathway has also been linked to the downregulation of adipogenic factors during the early stage of adipocyte differentiation. Thus, we examined the phosphorylation levels of MAPK signaling components in 3T3-L1 cells with or without *L. plantarum* HAC01 lysate. Treatment with *L. plantarum* HAC01 lysate prevented the phosphorylation of ERK, p38, and JNK in differentiated 3T3-L1 cells (Figure 8). In particular, ERK phosphorylation was the most strongly inhibited. These results suggest that the attenuation of MAPK signaling may contribute to the inhibitory effect of *L. plantarum* HAC01 lysate on adipocyte differentiation.

## 3. Discussion

Probiotics and probiotic lysates are beneficial for host nutrition and health, particularly with regard to the gut environment [19]. Previous work has reported on the beneficial effects of probiotic lysates on various diseases. Several studies have indicated that *Lactobacillus* lysates ameliorate colitis by strengthening gut barrier function and modulating the mucosal inflammatory system [8,20]. Moreover, *Lactobacillus* lysates enhance antitumor immune responses [21] and improve hypercholesterolemia [22]. Furthermore, previous studies have demonstrated that *L. plantarum* ATG-K2 lysate prevents adipocyte differentiation in vitro [23]. These results are in line with our finding that *L. plantarum* HAC01 lysate inhibits the differentiation of adipocytes, providing precedent for its use in treating obesity.

The differentiation of pre-adipocytes into mature adipocytes occurs in several stages: early, intermediate, and terminal [24]. C/EBPβ is an essential transcription factor involved in adipocyte differentiation that stimulates adipogenesis during the early stage by activating other major transcription factors, such as C/EBPα, PPARγ, and SREBP-1c [25]. Therefore, C/EBPβ is crucial for progression through the early stage of adipocyte differentiation and its inhibition by *L. plantarum* HAC01 lysate is an important mechanism underlying the anti-adipogenic effects of HAC01 (Figure 4). C/EBPα, PPARγ, and SREBP-1c also play critical roles in regulating adipogenesis [26]. SREBP-1c regulates fatty acid and triglyceride synthesis by stimulating the expression of ACC and FAS and inducing C/EBPα and PPARγ expression [27]. When C/EBPα and PPARγ are expressed, the size and number of lipid droplets increases and the lipid–protein pathway is upregulated, promoting adipocyte differentiation [28]. Additionally, PPARγ activation is upstream of aP2 and LPL expression in adipose tissue [29]. In this study, *L. plantarum* HAC01 lysate significantly suppressed the expression of aP2, LPL, ACC, and FAS during the terminal stage of differentiation (Figure 2 and Figure 3), and that of C/EBPα, PPARγ, and SREBP-1c during early and terminal stages (Figure 5 and Figure 6). Taken together, these results support the effectiveness of HAC01 lysate in inhibiting adipogenesis throughout the differentiation process.

AMPK is a key regulator of various physiological processes, including cellular energy homeostasis and lipid and fatty acid metabolism [29]. Increase in AMP activates AMPK and promotes ATP production, leading to suppress energy consumption, such as adipogenesis and lipogenesis [10,30]. Therefore, AMPK signaling is a targeted pathway to treat metabolic diseases, including obesity. When AMPK is phosphorylated, it becomes activated and suppresses the expression of adipogenic and lipogenic factors, such as C/EBPs, PPARγ, and SREBP-1c [30], which is also shown our results (Figure 2, Figure 3, Figure 4, Figure 5 and Figure 6). As seen in our results (Figure 3A), activated AMPK also promotes the phosphorylation and inactivation of ACC, which is important for mitochondrial fatty acid synthesis and consumption [31]. The effect of *L. plantarum* HAC01 lysate on AMPK phosphorylation was partially, but significantly, reversed by Compound C (Figure 7B), suggesting that AMPK may be directly involved in the *L. plantarum* HAC01 lysate-mediated inhibition of adipocyte differentiation.

MAPK signaling is also involved in the early differentiation of adipocytes. The MAPK signaling pathway is activated through phosphorylation by extracellular stimuli, inducing various intracellular responses [32]. ERK is phosphorylated by C/EBPβ, which is expressed during the early stage of differentiation, and induces the expression of C/EBPα and PPARγ to promote adipocyte differentiation [33]. In addition, p38 has been previously reported to regulate adipocyte differentiation during the early stage [34], and JNK has been reported to be both a positive and negative regulator of adipocyte differentiation [15,34,35,36]. In this study, we found that the phosphorylation of ERK, p38, and JNK was suppressed following treatment with *L. plantarum* HAC01 lysate (Figure 8), implicating the inhibition of MAPK signaling as a mechanistic component underlying the anti-adipogenic effect of HAC01 lysate.

**Figure 9:**
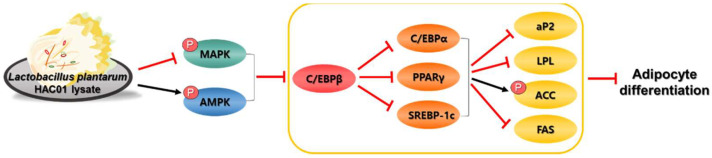
Schematic diagram of anti-adipogenic effects of *Lactobacillus plantarum* HAC01 lysate in 3T3-L1. *L. plantarum* HAC01 lysate suppresses on adipocyte differentiation the early stage of adipocyte differentiation via AMPK activation and MAPK inhibition. MAPK, mitogen-activated protein kinase; AMPK, adenosine monophosphate-activated protein kinase; C/EBP, CCAAT/enhancer-binding protein; PPARγ, peroxisome proliferator-activated receptor gamma; SREBP-1c, sterol regulatory element-binding protein 1c; aP2, adipocyte protein 2; LPL, lipoprotein lipase; ACC, acetyl-CoA carboxylase; FAS, fatty acid synthase.

In conclusion, this study demonstrated that *L. plantarum* HAC01 lysate inhibits lipid accumulation and gene expression of adipocyte differentiation and lipid synthesis factors. Moreover, this inhibition is facilitated via AMPK activation and MAPK inhibition (Figure 9). Owing to its ability to negatively regulate adipocyte differentiation, *L. plantarum* HAC01 lysate may be a promising anti-obesity agent. However, further studies are needed to confirm the effects of *L. plantarum* HAC01 lysate in animal models and humans.

## 4. Materials and Methods

### 4.1. Materials

A lysate of *L. plantarum* HAC01 was obtained from AtoGen (AtoGen Co., Ltd., Daejeon, Korea) and prepared as previously described [16,23]. 3T3-L1 pre-adipocytes were purchased from American Type Culture Collection (Manassas, VA, USA). Dulbecco’s modified Eagle’s medium (DMEM) and Dulbecco’s phosphate-buffered saline were purchased from Corning Inc. (Corning, NY, USA). Bovine calf serum, fetal bovine serum (FBS), antibiotic–antimycotic solution, 0.5% trypsin-ethylenediaminetetraacetic acid, and insulin were provided by Gibco BRL (Grand Island, NY, USA). Dexamethasone, 3-isobutyl-1-methylxanthine, paraformaldehyde, Oil Red O solution, and isopropanol were purchased from Sigma-Aldrich (St. Louis, MO, USA). Skim milk was purchased from BD Difco (Sparks, MD, USA). Primary antibodies were purchased from Abcam (Cambridge, UK), Cell Signaling Technology (Danvers, MA, USA), and Santa Cruz Biotechnology (Dallas, TX, USA) (Table S1).

### 4.2. Cell Culture and Differentiation

3T3-L1 pre-adipocytes were cultured in 10% bovine calf serum-DMEM in a 5% CO_2_ incubator at 37 ℃. To induce adipocyte differentiation, 3T3-L1 cells were seeded at a density of 0.5 × 10^5^ cells/mL and maintained for 2 d. Pre-adipocytes were then induced with a differentiation medium comprising 10% FBS-DMEM with an MDI cocktail (500 μM 3-isobutyl-1-methylxanthine, 1 μM dexamethasone, and 10 μg/mL insulin) on day 0. On day 3, the differentiation medium was replaced with 10% FBS-DMEM containing insulin. On day 5, cells were maintained in 10% FBS-DMEM up to day 7. To evaluate the effects of *L. plantarum* HAC01 lysate on adipocyte differentiation, cells were cultured in a differentiation medium with varying concentrations of *L. plantarum* HAC01 lysate (0, 50, 100, 200, and 400 μg/mL).

### 4.3. Cell Viability Assay

Cell viability assays were conducted using a Cell Counting Kit-8 (CCK-8; Dojindo Laboratories, Kumamoto, Japan) according to the manufacturer’s instructions. 3T3-L1 cells were seeded at a density of 1 × 10^5^ cells/mL for 24 h and treated with varying concentrations of *L. plantarum* HAC01 lysate (0, 25, 50, 100, 200, and 400 μg/mL). After incubation for 72 h, CCK-8 solution (10 μL) was added to each well and cells were incubated for 2 h in the dark. The absorbance was measured at 450 nm using an Epoch microplate spectrophotometer (BioTek Instruments, Winooski, VT, USA).

### 4.4. Oil Red O Staining

Differentiated 3T3-L1 cells were fixed in 4% paraformaldehyde for 1 h and washed with distilled water. The cells were then stained with Oil Red O solution for 30 min. After removing the staining solution and washing four times with distilled water, images of the stained lipid droplets were obtained using an ECLIPSE Ts2 microscope (Nikon, Tokyo, Japan). The dye retained in the cells was eluted with 100% isopropanol and quantified by measuring the absorbance at 500 nm using an Epoch microplate spectrophotometer (BioTek Instruments, Winooski, VT, USA).

### 4.5. Quantitative Reverse-Transcription Polymerase Chain Reaction (qRT-PCR)

mRNA expression was measured using qRT-PCR. In brief, total RNA was extracted using the easy-spin ^TM^ Total RNA Extraction Kit (iNtRON Biotechnology, Seongnam, Korea) and 50 ng/μL was measured using a Take3 Micro-Volume plate (BioTek Instruments, Winooski, VT, USA). Complementary DNA was synthesized using GoScript Reverse Transcriptase (Promega, Madison, WI, USA). qRT-PCR was performed using a QuantStudio 3 real-time PCR instrument (Thermo Fisher Scientific, Waltham, MA, USA). All genes were normalized to β-actin. Sequences are provided in Table S2.

### 4.6. Western Blotting

Differentiated 3T3-L1 cells were lysed in PRO-PREP™ Protein Extraction Solution (iNtRON Biotechnology, Seong-nam, Korea) supplemented with a phosphatase inhibitor (Thermo Fisher Scientific, Waltham, MA, USA). Following quantification, 40 μg of protein sample was loaded on a 10% sodium dodecyl sulfate-polyacrylamide gel and transferred onto a polyvinylidene difluoride membrane. After blocking with 5% skim milk for 1 h at room temperature, the membranes were incubated with primary antibodies for 2 h, followed by horseradish peroxidase-conjugated secondary antibodies (Promega, Madison, WI, USA) for 1 h. Protein bands were visualized using the ImageQuant ^TM^ LAS 500 system (GE Healthcare, Upsala, Sweden).

### 4.7. Statistical Analysis

All data are presented as the mean ± standard deviation of at least three independent experiments. Statistical significance was analyzed using a one-way analysis of variance (ANOVA), followed by Tukey’s post hoc tests using GraphPad Prism 9 software (Graph Pad Software Inc., San Diego, CA, USA). Results with *p* < 0.05 were considered statistically significant.

## Data Availability

The experimental data are available on request by corresponding author.

## Supplementary Materials

The following supporting information can be downloaded at: https://www.mdpi.com/article/10.3390/ijms23115901/s1.
