# Supplementary material for: Ameliorative Effects of Lactobacillus plantarum HAC01 Lysate on 3T3-L1 Adipocyte Differentiation via AMPK Activation and MAPK Inhibition
Source: Int J Mol Sci. 2022 May 24;23(11):5901. doi: 10.3390/ijms23115901 (PMC9180524; doi:10.3390/ijms23115901)
Supplement: Supplementary file 1 [file ijms-23-05901-s001.zip › ijms-1738887-supplementary.pdf]

Table S1. Antibodies for western blotting.

Antibody	Dilution	Supplier	Catalog No.
p-ACC	1:1000	Abcam	ab68191
ACC	1:1000	Cell Signaling Technology	#3662
FAS	1:1000	Santa Cruz Biotechnology	sc-48357
C/EBPβ	1:1000	Cell Signaling Technology	#3087
C/EBPα	1:1000	Cell Signaling Technology	#8178
PPARγ	1:1000	Cell Signaling Technology	#2443
SREBP-1c	1:500	Santa Cruz Biotechnology	sc-13551
p-AMPK	1:1000	Cell Signaling Technology	#2535
AMPK	1:1000	Cell Signaling Technology	#2532
p-ERK	1:1000	Cell Signaling Technology	#4370
ERK	1:1000	Cell Signaling Technology	#4695
p-p38	1:1000	Cell Signaling Technology	#4511
P38	1:1000	Cell Signaling Technology	#8690
p-JNK	1:1000	Cell Signaling Technology	#4668
JNK	1:1000	Cell Signaling Technology	#9252
β-actin	1:1000	Abcam	ab6276

ACC, acetyl-CoA carboxylase; FAS, fatty acid synthase; C/EBP, CCAAT/enhancer-binding protein; PPARγ, peroxisome proliferator-activated receptor gamma; SREBP-1c, sterol regulatory element-binding protein 1c; AMPK, adenosine monophosphate-activated protein kinase; ERK, extracellular regulated kinase; JNK, c-Jun -N-terminal kinase.

Table S2. Primer sequences used for qRT-PCR.

Gene	Forward (5′-3′)	Reverse (5′-3′)
Acc	GCGTCGGGTAGATCCAGTT	CTCAGTGGGGCTTAGCTCTG
Fas	TTGCTGGCACTACAGAATGC	AACAGCCTCAGAGCGACAAT
Lpl	TCCAAGGAAGCCTTTGAGAA	CCATCCTCAGTCCCAGAAAA
aP2	TCACCTGGAAGACAGCTCCT	AATCCCCATTTACGCTGATG
C/ebpα	TTACAACAGGCCAGGTTTCC	GGCTGGCGACATACAGTACA
Pparγ	TTTTCAAGGGTGCCAGTTT	AATCCTTGGCCCTCTGAGAT
Srebp-1c	TGTTGGCATCCTGCTATCTG	AGGGAAAGCTTTGGGGTCTA
β-actin	CCACAGCTGAGAGGAAATC	AAGGAAGGCTGGAAAAGAGC

Acc, acetyl-CoA carboxylase; *Fas*, fatty acid synthase; *Lpl*, lipoprotein lipase; *aP2*, adipocyte Protein 2; C/EBPα, CCAAT/enhancer-binding protein alpha; PPARγ, peroxisome proliferator-activated receptor gamma; SREBP-1c, sterol regulatory element-binding protein 1c.
